# Managing school interaction networks during the COVID-19 pandemic: Agent-based modeling for evaluating possible scenarios when students go back to classrooms

**DOI:** 10.1371/journal.pone.0256363

**Published:** 2021-08-18

**Authors:** Ana María Hernández-Hernández, Rodrigo Huerta-Quintanilla

**Affiliations:** Departamento de Física Aplicada, Centro de Investigación y de Estudios Avanzados del Instituto Politécnico Nacional, Mérida, Yucatán, México; Post Graduate Institute of Medical Education and Research, INDIA

## Abstract

The most unexpected and toughest phenomenon that has occurred in recent times is the global COVID-19 pandemic. One of the first measures to prevent the spread of the disease was to close educational institutions. The students were forced to start a learning process through social networks and web platforms. In some countries, a return to face-to-face classes was established. However, weeks later, some of them had to return to virtual activities due to an upswing in the COVID-19 cases. In Mexico, classes have been held virtually, with face-to-face activities only re-established in two of the 32 states. In our state, Yucatan, scholarly activities are still virtual. In this work, the dispersion of COVID-19 at different academic establishments in Yucatan was simulated. Networks of Friendship, noncordial treatment, family ties and study groups were considered. Based on these networks, we evaluated the possibility of returning to school without inducing a rebound in the COVID-19 cases in the state. Agent-based simulations were used, with each student as an agent. Interaction rules were established based on international research regarding good practices in times of COVID-19. We used seven networks from different academic institutions, ranging from primary through college level. As a result, possible contagion curves were obtained for different scenarios, which leads to a discussion about the measures that would be relevant once a return to face-to-face classes is overseen. Simulations show that isolating students and reducing the number of students in the same classroom are good strategies and substantially reduce the possible contagiousness.

## Introduction

Since COVID-19 was first discovered in December 2019 in the province of Wuhan in China, humanity has had to quickly change the way people interact, in addition to suffering an increased economic and social crisis [1–4]. At the beginning of 2020, COVID-19 cases began to appear in Europe, America, and Africa. Very quickly, scientists from many disciplines began research to understand the virus and its spreading dynamics and tried to mitigate the global pandemic, undertaking different approaches [5–12]. Once the gravity of the situation was understood, governments from many countries decreed generalized lockdown to hold the spreading of the disease and to minimize the number of deaths due to COVID-19. Several clinical studies began to be conducted in China and in other countries of the world in which COVID-19 first appeared [9, 13]. Some studies were focused on transmission due to social interactions [4, 10, 11, 14], survival of the virus on surfaces [15], transmission on kids and teenagers [16–18], transmission in closed places [12, 19–21], and computerized simulations [22–24]. Complementary studies were focused on preventive measures and the evaluation of the impact of wearing protective masks [25–27]. By the end of February, the first case of COVID-19 arrived at the American continent, while cases began to rise in Europe and a contingency was declared in several countries. By the third week of March, in several states of Mexico, a lockdown of the population was imposed to hold back the virus’s spread and avoid a saturation of the health system. One of the first actions imposed in Mexico (and in many countries of the world) was the cessation of face-to-face classes, along with developing more online learning processes through social networks and virtual meeting platforms. In countries where the number of people developing COVID-19 went down, a gradual opening of daily activities began: people returned to work, recreation centers were opened, and even students returned to classrooms [28–30]. In contrast, some countries kept face-to-face classes and have been able to mitigate the spread of the virus in the population [31]. In the case of Yucatan State, face-to-face classes were suspended on March 20, 2020. From that week on, a lockdown was established throughout the state. Due to the proximity of a new school semester, it is of interest to evaluate the relevance and challenges that a return to face-to-face classes would represent in the state of Yucatan.

## Materials and methods

### School networks

Seven school networks were obtained through a survey in different academic establishments in Yucatan, which were used through a web platform before the pandemic lockdown. This study obtained ethics permission from the Bioethics Committee for Research on Humans COBISH—CINVESTAV (Comité de Bioética para la investigación en seres Humanos, COBISH—CINVESTAV). We obtained ethical permission from the committee in February 2019 with reference number 053/2018. Once the study was approved, we had talked with authorities from different academic institutions. We asked directors for their written acceptance to survey the students. At the moment of the survey, the students could decline or give their approval to save their answers. If a student agreed, the student signed an informed consent electronically at the end of the survey. The data acquisition started in March 2019 and ended in March 2020 because of the lockdown. We considered different types of relations such as friendship, unfriendly treatment, family ties (such as siblings and cousins), and study group partners. It is important to emphasize that all of the data collected through the survey followed the ethical requirements. To obtain the data, 4784 students from seven different schools were involved in the study. From the total number of students, 4323 students responded to the survey electronically through a web page saved on a server at Cinvestav Merida. At least 15 students were surveyed. Each group of 4 students has a surveyor as supervisor to avoid interactions between them. The response rate of the study was 86.45%. The survey used for obtaining the networks is in the S1 File. The adjacency matrices of the networks used in this study are in the S2 File. Once obtained, data were cleaned considering the following different aspects: 1) friendship and unfriendly treatment are mutually exclusive 2) siblings and cousins relationships are mutually exclusive 3) cousins of my brothers/sisters are my cousins, 4) the brothers/sisters have the same last names, 5) the cousins have at least one of their last names in common and 6) the links of brothers/sisters and cousins are by definition bidirectional. The data of the students who did not fill out the survey or filled it out incompletely were removed. After all of the data cleaning, this study considered the data related to 4136 students from the seven different schools. Once we obtained the directed networks of friendship, unfriendly treatment, siblings, cousins, and study groups, a single weighted network was generated. The weights of the different links were assigned while accounting for two things: 1) the number of relationships that students have between them (for example, being friends and also cousins), and 2) how close they can be due to each relationship (for example, a friend is closer than a group study partner). A weight of 0.5 was assigned to friends, while accounting for the fact that students tend to spend as much time as possible with their friends. In the case of unfriendly treatment, a weight of 0.1 was assigned given that the students would try to avoid a person with whom they do not get along, but there will still be compulsory interactions. The brother/sister link is assigned a weight of 0.5 because students and their brothers/sisters share space and time in family activities. Links associated with cousins weighted 0.25, as they could share time outside the school regularly but less extensively than with siblings. Finally, the links associated with the study groups obtained a weight of 0.2 since the interaction is mainly due to an academic need rather than a voluntary desire to share time with the partner. The assigned weight is normalized to have a value between 0 (there is no relationship) and 1 (they have as many relationships as possible). Table 1 shows the weight of each of the different link types present in the network and their respective normalization. Once weighted networks were established, they were incorporated into agent-based simulations to simulate the spread of COVID-19 in each school network.

**Table 1:** Weighted links. The links weight is associated with the number and type of links between nodes. The last column shows the normalization of the assigned link weight.

Relation type	Assigned link weight	Normalized link weight
Friends	0.5	0.42
Foes (Unfriendly treatment)	0.1	0.08
Brothers/Sisters	0.5	0.42
Cousins	0.25	0.21
Small group study partners	0.2	0.17
Friends and Brothers/Sisters	1	0.83
Friends and Cousins	0.75	0.63
Friends and Small group study partners	0.7	0.58
Foes and Brothers/Sisters	0.6	0.5
Foes and Cousins	0.35	0.29
Foes and Small group study partners	0.3	0.25
Brothers/Sisters and Small group study partners	0.7	0.58
Cousins and Small group study partners	0.45	0.38
Friends, Brothers/Sisters and Small group study partners	1.2	1
Friends, Cousins and Small group study partners	0.95	0.79
Foes, Brothers/Sisters and Small group study partners	0.8	0.66
Foes, Cousins and Small group study partners	0.55	0.46

### Spread of COVID-19 in school networks

#### Variables considered in the simulations

**Fig 1:**
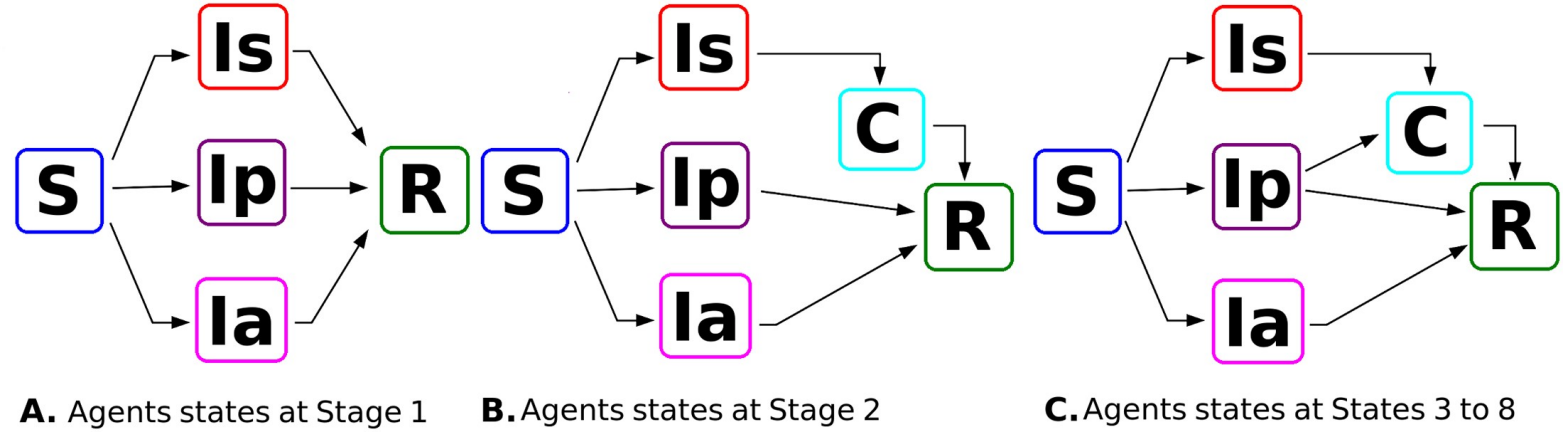
Possible states of the agents throughout the simulation. Susceptible S, symptomatic Is, presymptomatic Ip, asymptomatic Ia, and recovered R. Since Stage 2, we also have the Confined state C.

*Agents status*. From scientific reports on COVID-19 (which are numerous and impossible to review in their entirety) and from reports of both the WHO and country authorities, we established the determinant factors when studying the spread of COVID-19 in school facilities [5, 17, 32, 33]. The first variable established for the model was the agents’ status, with the possibility of being symptomatic, presymptomatic, or asymptomatic (Fig 1). These states were based on the bibliography that called attention to those individuals who had little or no symptoms, who could remain to infect their peers for several days before being detected and confined [7, 32, 34]. The number of patients who could be asymptomatic or presymptomatic has not been globally established. Some publications indicate various percentages, between 10 and 80%, and in some cases, the values discussed come from computerized simulations [35]. Here, we evaluate the effects of having different percentages of presymptomatic and asymptomatic agents. Then, the possible statuses for agents are the following: Susceptible (S), Symptomatic (Is), Presymptomatic (Ip), Asymptomatic (Ia) and Recovered (R). In later simulations, agents can also take the status of Confined (C).

*Variables included in the interaction rules*. To determine a form of the interaction rules, a bibliographic review was conducted to identify the relevant variables. The variables considered in this work are listed below:

1. State of health: Different studies show that the general state of health can be relevant, because the severity of symptoms of COVID-19 reached by a patient can depend on comorbidities and previous conditions. These issues can make the individual more prone to acquire the virus if there exists a low immune response, and individuals could carry symptoms that can worsen and even lead to death [36, 37].
2. Closed spaces: Several studies conducted in confined spaces showed that it is more likely to acquire the virus if people interact with an infected person inside an enclosed space, especially if it does not have adequate ventilation [12, 19, 20].
3. Adoption of hygiene standards: Many studies have shown that adopting hygiene measures such as hand washing, disinfection of high traffic areas, and the use of face masks reduces the possibility of contagion [5].
4. Physical distance: COVID-19 is present in saliva, according to various studies [38–40]. Then, when we speak, microdroplets of the saliva of different diameters are expelled. These droplets can reach an interlocutor and infect him, which is why distancing measures have been established in a large number of places.
5. Social proximity: In this case, it is assumed that the interactions that lead to a greater probability of contagion are those between people who have a relationship [4, 41]. Thus, social proximity is the weight of the link in the network. This weight is associated with the number and type of relationships between two network agents.
6. Viral load: This variable is related to the moment that the symptoms can be observed. The viral load is considered in the most advanced part of the simulations [6, 34].

Based on these variables, we established an interaction rule between agents that allows determining how possible it is for an agent to infect its neighbors. A person who has more in-links will be more likely to be infected. Similarly, if someone has a large number of out-links, they can infect more people. The forms that the interaction rule takes are detailed in the subsection on interaction rules.

#### Simulation details

Using agent-based simulations, a series of stages were established to evaluate a return to face-to-face classes in Yucatan, in light of the type of conditions that would be required to make it possible.

*Agent attributes*. Along with the simulations, agents have different attributes, which were selected based on an extensive amount of literature. These values were established to introduce heterogeneity in agent properties and interactions. It is important to note that those values can change if those are required for future simulations and new findings. Initially, agents have the following attributes:

1. Age
2. Sex
3. Course
4. Status: the node is Susceptible (S), Symptomatic (Is), Presymptomatic (Ip), or Asymptomatic (Ia). Initially, everyone except for one agent is S. The infection status occurs in the Ip, Ia and Is states. In later simulations, the agents can also take the status Confined (C).
5. General health condition: Based on data from the state of Yucatan [42], it is assumed that 86% of infants and young people have good health (value 0.0), 8% have little compromised health (value 0.5), and 6% have a precarious state of health (value 1.0). Once the agent goes from state S to an infected status (Ia, Ip, or Is), the value of the health condition attribute changes, taking a value of zero for the Ia case, a value assigned randomly in a uniform distribution between 0.1 and 0.8 for Ip agents, and a value of 1.0 for Is cases.
6. Symptoms: For agents in the susceptible state, the symptoms attribute has a value of zero. For agents that change their state to Ip, the symptoms attribute takes a random value in a uniform distribution U (0.1,0.85). For those agents who reach the Is state, their symptoms attribute will take a value greater than 0.85, and for those who go to the Ia state, it will take a value of zero.
7. Having a sick relative: If an agent does not have a sick relative at home, the value of this attribute is zero. If the agent has a sick relative who does not live in the same house, it takes a value of 0.5, because there could be an occasional contagion. If there is a sick relative at home, then the value of this attribute is equal to one. Any of these three values are assigned randomly in the simulation.
8. Adoption of hygiene standards: This attribute simulates how much the agent adopts hygiene standards for the prevention of COVID-19 contagion (washing hands, avoiding direct physical contact, use of face masks, and so on). It takes a value of 1 if the agent does not adopt any hygiene rules, or -1 if all hygiene rules are adopted. This variable is set randomly from a uniform distribution between -1 and 1.
9. Possibility of infection (associated with the viral load): This attribute is based on curves obtained in different clinical studies used as a guide to establish the functional form of our curves [6]. The viral load profile was incorporated in the latest stages of simulations and was contemplated depending on the symptoms shown by the agent.
10. Days of transition to recovery: Once the agent goes from state S to any state of infection (Ia, Ip, or Is), the agent acquires an attribute that indicates how many days it will be convalescing until a recovery state is reached. For asymptomatic agents, this attribute takes a value of 14 days; for presymptomatic agents, between 14 and 18 days; and for symptomatic cases, it takes values between 18 and 22 days [6].

The simulations were run for 100 time steps (equivalent to 100 days), and 300 repetitions were performed.

*Interaction rules*. In this section, we establish the interaction rules for the different simulations. In the first interaction rule, variations in the viral load are not weighed. It is given by

$$\begin{array}{c} pc=\frac{1}{5}*\left(c+es+h+df+fen\right)*ps \end{array}$$ (1)

where *pc* is the possibility of infecting a contact, *c* is the possibility of contagion in a closed space, *es* is the possibility that the agent becomes infected given that its state of health is accounted for, *h* is the possibility that the contact becomes infected depending on how well it adopts hygiene standards, *df* is the physical distance between the contact and the infected agent (its value is given by the linear relationship *df* = 1.02 − 0.68x), *fen* is the possibility of becoming infected if the agent has a sick relative, and *ps* is the social proximity. An average of the variables determinant in the infection process was performed to assure the accuracy of the study.

Furthermore, we developed an interaction rule that accounts for the viral load profiles of the infected node. In that case, the interaction rule is

$$\begin{array}{c} pc=\frac{1}{6}*\left(c+es+h+df+fen+pcv\right)*ps \end{array}$$ (2)

where *pcv* is the possibility that the infected agent infects its contacts depending on the evolution of the disease. The *pcv* value varies with time (each day of convalescence) and is approximated by the distribution,

$$\begin{array}{c} Pc\left(t\right)=\frac{1}{Pc(ds)}\frac{ds^{t}e^{-ds}}{t!} \end{array}$$ (3)

where *Pc*(*t*) is the possibility of infecting on day t, *Pc*(*ds*) is the possibility of infecting on the day ds on which the agent presents the symptoms or has the highest viral load [6]. The value of ds depends on the status of the infected agent. For Is agents, it is between 1 to 3 days, 2 to 4 for Ip, and 4 to 6 for Ia. Day t ranges from 0 to 14 days for asymptomatic agents, 0 to 18 for presymptomatic agents, and 0 to 22 days for symptomatic agents.

*Possibles scenarios*. In this study, different stages are proposed in the simulation to evaluate the possible scenarios of the spread of the virus in school networks given different considerations.

- Stage 1:
  At this stage of the simulation, all infected agents can infect during all of the days of their convalescence. This scenario would correspond to taking no action without considering that the patient could die. At the end of the recuperation time, each agent will go to a recovery state. The percentage of agents in states Is, Ip, and Ia is modified. It is established that 10 to 90% of the agents could be in another infected state other than Is. From this percentage, 15 to 90% could be in the Ia condition.
- Stage 2:
  Is patients are confined, and each Is agent will have one day to infect its peers before it is confined. Ia and Ip patients will not be detected and could continue to infect during the days in which they remain in this state.
- Stage 3:
  In this case, Ip patients could increase their state of symptoms until their symptoms attribute is high enough (they reach a value of 0.9) to change their state to Is. This rule means that agents in state Is and some agents initially in state Ip will be confined during the simulation. At each time step, the symptoms attribute of the Ip agents will increase by 0.05. If an Ip agent reaches the value of 0.9, it will become Is, and then, it will be isolated for the remaining time of its convalesce. Ia agents will continue to infect every day until they are considered to be recovered agents.
- Stage 4:
  Stage 4 is functionally the same as stage 3. However, the agents that are established as siblings of a symptomatic agent are confined at the same time as the infected agent. In the case of Ip patients who reach the Is state, his/her siblings are also confined at the same time, whether his state is S, Ip or Ia.
- Stage 5:
  In this stage and the next stages, the viral load profile is incorporated, with the interaction rule given by Eq 2. The confinement strategies implemented in the previous stages are still considered.
- Stage 5A: In this stage, the nodes (agents) in the networks are selected to be in an immunized state. There are four ways to choose an agent to be immunized: 1) randomly, 2) by its degree, 3) by its clustering, and 4) by its betweenness. The degree provides a size that reflects how popular a node is in the network. The clustering measures how well connected are the neighbors of an agent among themselves. The betweenness measures how important a node is for the network interconnections.
- Stage 6A: The classrooms are separated into smaller groups of no more than 20 agents, with all of the links being maintained.
- Stage 6B: The classrooms are isolated. In this case, the links between students from different classrooms are removed except for cousins or siblings’ links, because they can probably interact outside the school.
- Stage 7: In this scenario, stages 6A and 6B are combined. The classrooms are divided into groups of no more than 20 agents, and the groups are isolated. Only the sibling and cousin links between agents from different groups are conserved. The conditions of all of the previous stages (isolation of symptomatic patients, isolation of siblings, contagion profiles, and so on) are kept in this stage.
- Stage 8: Because some students can get infected in other circumstances outside of family or student partner interactions, we give a daily probability that a randomly chosen agent gets infected. At this stage, we simulate how these newly infected agents impact the spreading of the virus, depending on the daily probability of their appearance. For this stage, we run the simulation for 250 time steps.

**Fig 2:**
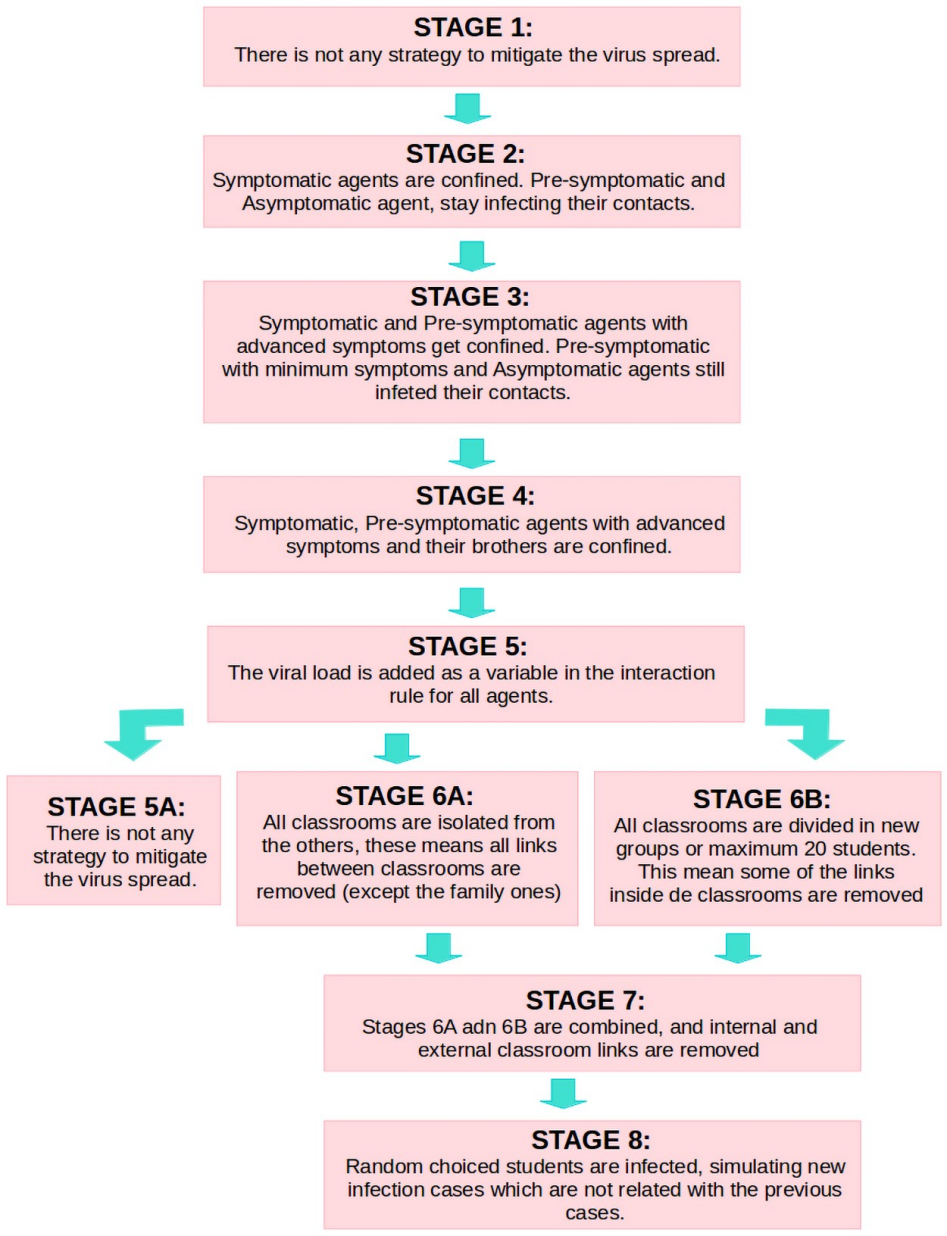
Different stages of the simulations.

A summary of the different stages for our simulations is shown in Fig 2.

## Results

Seven school networks, Sch1, Sch2, Sch3, Sch4, Sch5, Shc6, and Sch7, were obtained through surveys conducted through a web platform. The number of links and nodes and the characteristics of the weighted networks obtained are shown in Table 2. The networks obtained in this study have properties similar to those found by Huerta-Quintanilla et al [43]. These networks were introduced into agent-based simulations to determine how COVID-19 would spread in each of them.

**Table 2:** Characteristics of school-directed networks. Networks of friendship, noncordial treatment, siblings, cousins, and study groups were characterized. Additionally, these networks were used to build the weighted network employed on the simulations. The type of school could be **E**lementary/**S**econdary/**H**igh School/**Un**iversity. Additionally, it can be located in an **U**rban area or a **R**ural area.

Characteristics	Sch 1	Sch 2	Sch 3	Sch 4	Sch 5	Sch 6	Sch 7
Type of school	E	S	S	S	H	H	Un
Location	U	R	U	U	U	R	R
Nodes	562	270	456	613	1497	74	664
Friends Links	3350	1523	2840	2994	11618	367	3583
Foes Links	794	442	720	829	1301	94	544
Brothers/Sisters Links	150	26	50	62	170	12	44
Cousins Links	208	140	162	324	212	18	112
Small Study Group Links	467	541	758	1083	4089	169	1583
Directed mix weighted Links	4586	2220	3890	4526	14292	516	4628
Is it strongly connected?	No	Yes	No	No	No	Yes	No
Is it weakely connected?	Yes	Yes	Yes	Yes	Yes	Yes	Yes
<kou*t*>, <kin>	8.16	8.23	8.53	7.38	9.54	6.97	6.97
Density	0.014	0.03	0.019	0.012	0.006	0.095	0.01
Networks diameter	-	8	-	-	-	7	-
Average shortest path length	4.03	3.51	3.64	4.04	4.29	2.85	4.43
Average clustering	0.19	0.29	0.25	0.2	0.22	0.32	0.29
Average node betweenness	0.0054	0.0093	0.0058	0.005	0.0022	0.0257	0.0052
Average link betweenness	0.0008	0.0015	0.0009	0.0008	0.0003	0.0055	0.0009

The value of *R*_0_ (stablish as the basic reproduction number which is associated with the number os secondary cases) was calculated for each network imposing stage 1: all infected nodes can infect others during the convalescence period. *R*_0_ was obtained by counting the secondary infections of each infected node. The secondary infections are then averaged over infected nodes and repetions in the time steps in which the curve rises exponentially (the first 10 time steps, in our case). Table 3 shows the values of *R*_0_ obtained for each network. For COVID-19 different researchers find multiple values of *R*_0_ between 1.3 and 3.5 [2, 24, 44, 45].

**Table 3:** *R*_0_ calculated for each school network. The value of *R*_0_ was based on the secondary cases. This operation accounted for the infected agents, contacts infected, and agent out-degree. Additionally, the model included the interval of time when the infected curve was growing to avoid a subestimation due to a low number of susceptible agents. The registered value is an average over the infected nodes and the repetitions.

Educational Institution	Ro
Sch1	2.9
Sch2	2.8
Sch3	2.8
Sch4	3.1
Sch5	2.4
Sch6	3.2
Sch7	3.3

**Fig 3:**
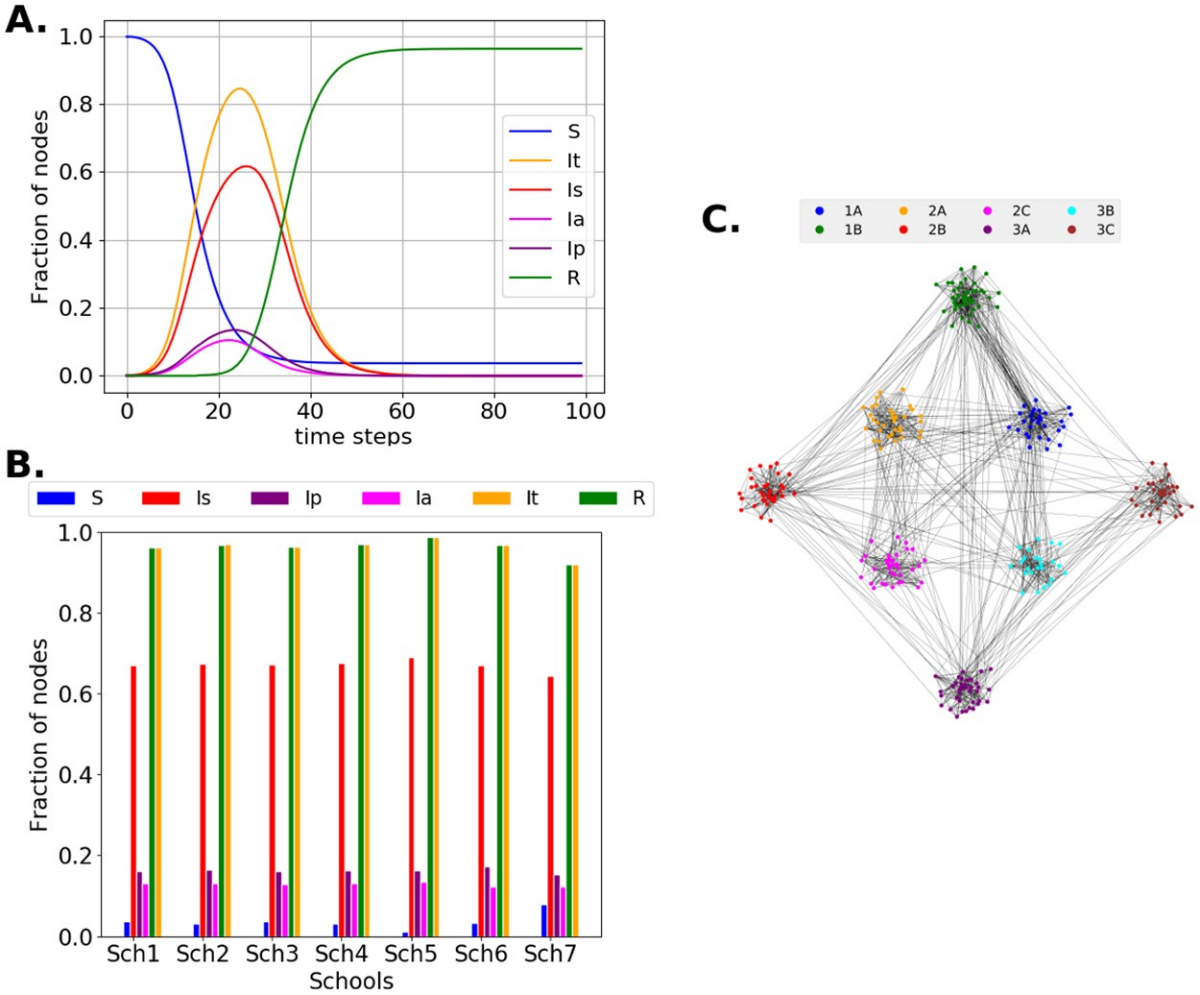
**A**. Stage 1 for Sch4. Blue S, red Is, purple Ip, magenta Ia, orange fraction of nodes infected with any type of symptom, and green R agents. **B**. All school networks in stage 1: the accumulated percentages of agents at each state are shown for every school. The results shown in **A** and **B** have 30% of agents without symptoms, from which 45% are asymptomatic. C. Sch2 network obtained. The nodes are classified by classrooms.

The characteristic curves (S, Is, Ip, Ia, and R) are shown in Fig 3A. Additionally, at stage 1, it is visible that the behavior of the virus spread is similar in all of the school networks. Therefore, the subsequent results will depend on the differences between the stages and between the different percentages of the Ip and Ia agents present in the network, as in Fig 3B. Additionally, the Sch2 network is shown in Fig 3C.

**Fig 4:**
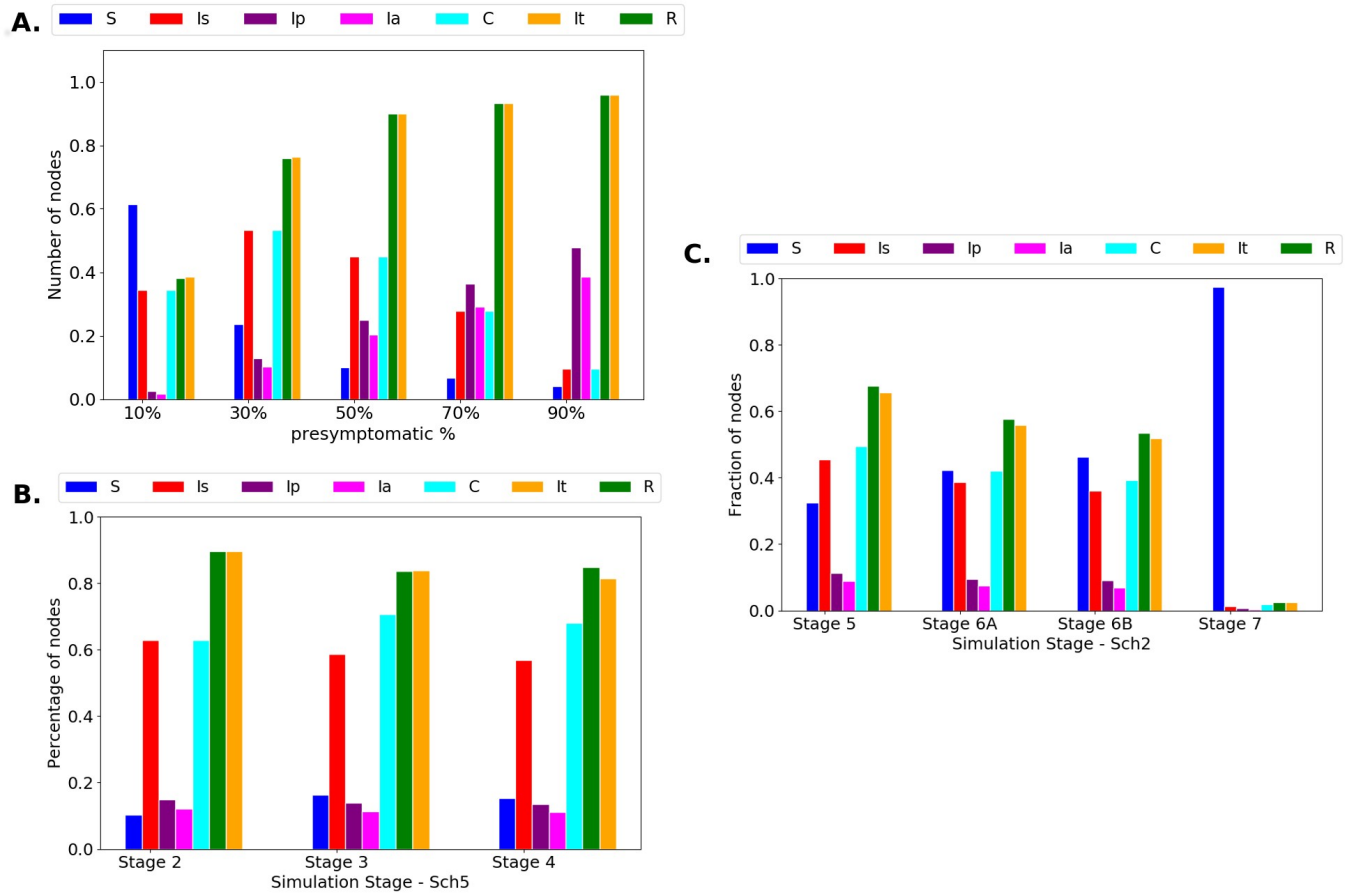
**A**. Sch7 on Stage 2. In this case, the percentage of nodes (or agents) on states Ip and Ia increases, and agents in state Is are confined. Then, the spread of the disease increases due to agents in states Ip and Ia that are not detected and confined. **B**. Comparing stages 2, 3 and 4 on Sch5 network: In this case, the figure shows the results obtained for Sch5 at each stage (1 to 4). In these cases, 30% of the infected students are not symptomatic, and from these, 45% are asymptomatic. **C**. Comparing stages 5, 6A, 6B and 7 for Sch2, 30% of the agents were not symptomatic, and from these, 45% were asymptomatic.

Fig 4A shows an increment of infected agents when the number of presymptomatic and asymptomatic agents changes in the simulations. A comparison among stages 2, 3, and 4 is shown in Fig 4B. It is evident that there is no significant decrease in the percentage of infected agents (less than 5%) when Ip agents achieve high symptoms and become confined (stage 3). Moreover, siblings’ confinement does not produce any relevant decrease in the total infected cases.

Fig 4C shows a comparison among stages 5, 6A, 6B and 7. In this stages, the viral load is considered in the simulation using Eqs 2 and 3 (Methods Section). From the results, it can be observed that the division of the classrooms in small groups (stage 6A) produces a decrease in the total number of infected agents (a decrease of 15.2% in the total number of infected agents compared to Stage 5). Likewise, the isolation of classrooms contribute to the decrease of infected agents (a decrease of 21.0% in the total number of infected agents compared to Stage 5). However, the best scenario occurs when classrooms are divided into smaller groups, and additionally these small groups are isolated. At Stage 7 the number of total infected decreased in 96.3% compared to Stage 5. The high diminishing of infected agents is originated in a modification of the network: around 50% of the links present in the classrooms and the links between students of different classrooms are removed. This removal means that the networks are modified and transformed into disconnected networks conformed by several clusters.

**Fig 5:**
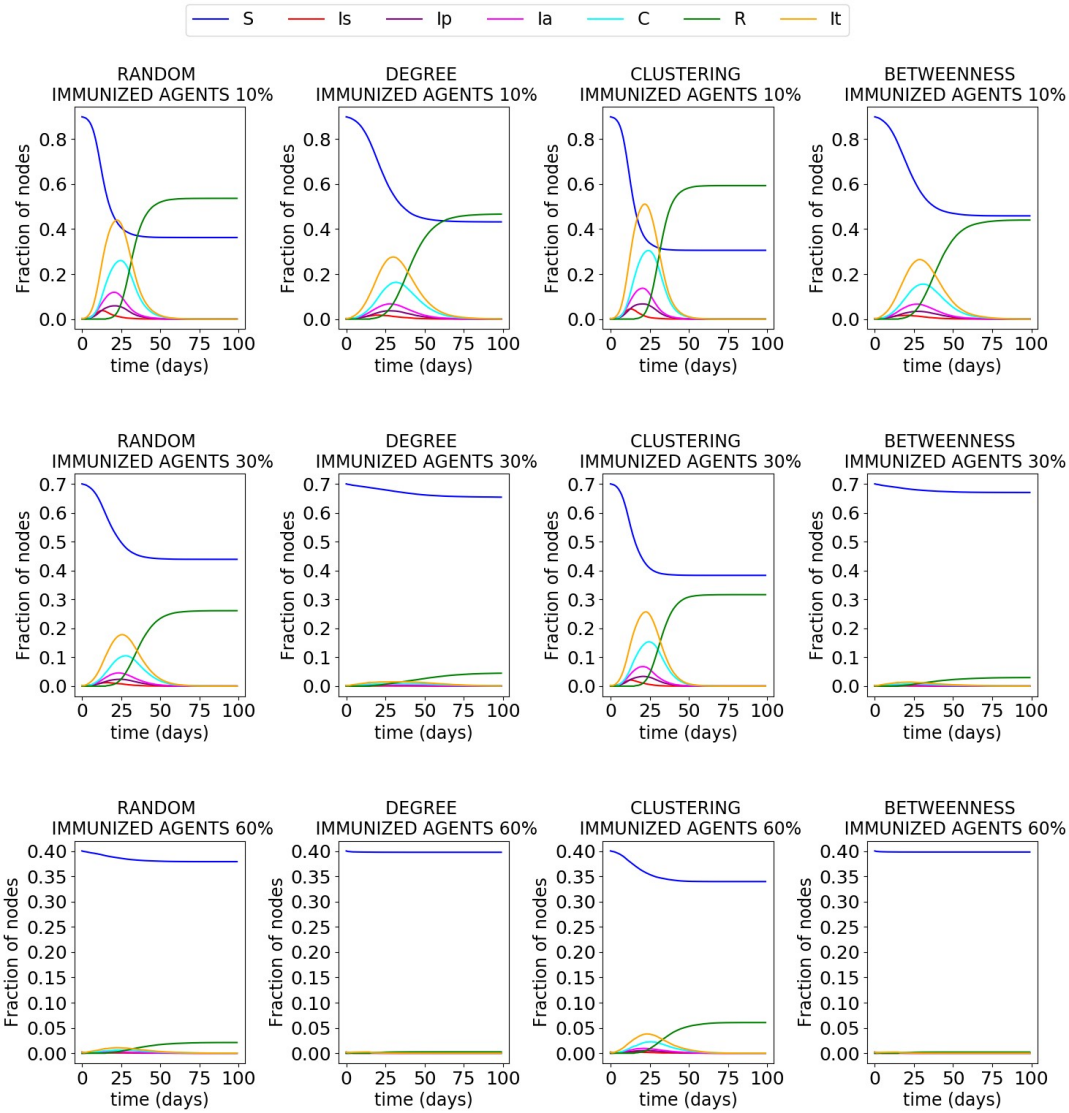
Introducing a new presymptomatic agent with a daily random probability.

The interaction rules developed in this work contemplate the probability of contagion from an infected family member. Yet, a student may get sick in other places like restaurants, buses, reunions, etc. To deal with the existence of infected people outside of the schools, a daily probability of infection of a random agent is generated in stage 8. Fig 5 shows the results for daily probability of infection with values 0.1, 0.3, 0.5, and 0.7.

**Fig 6:**
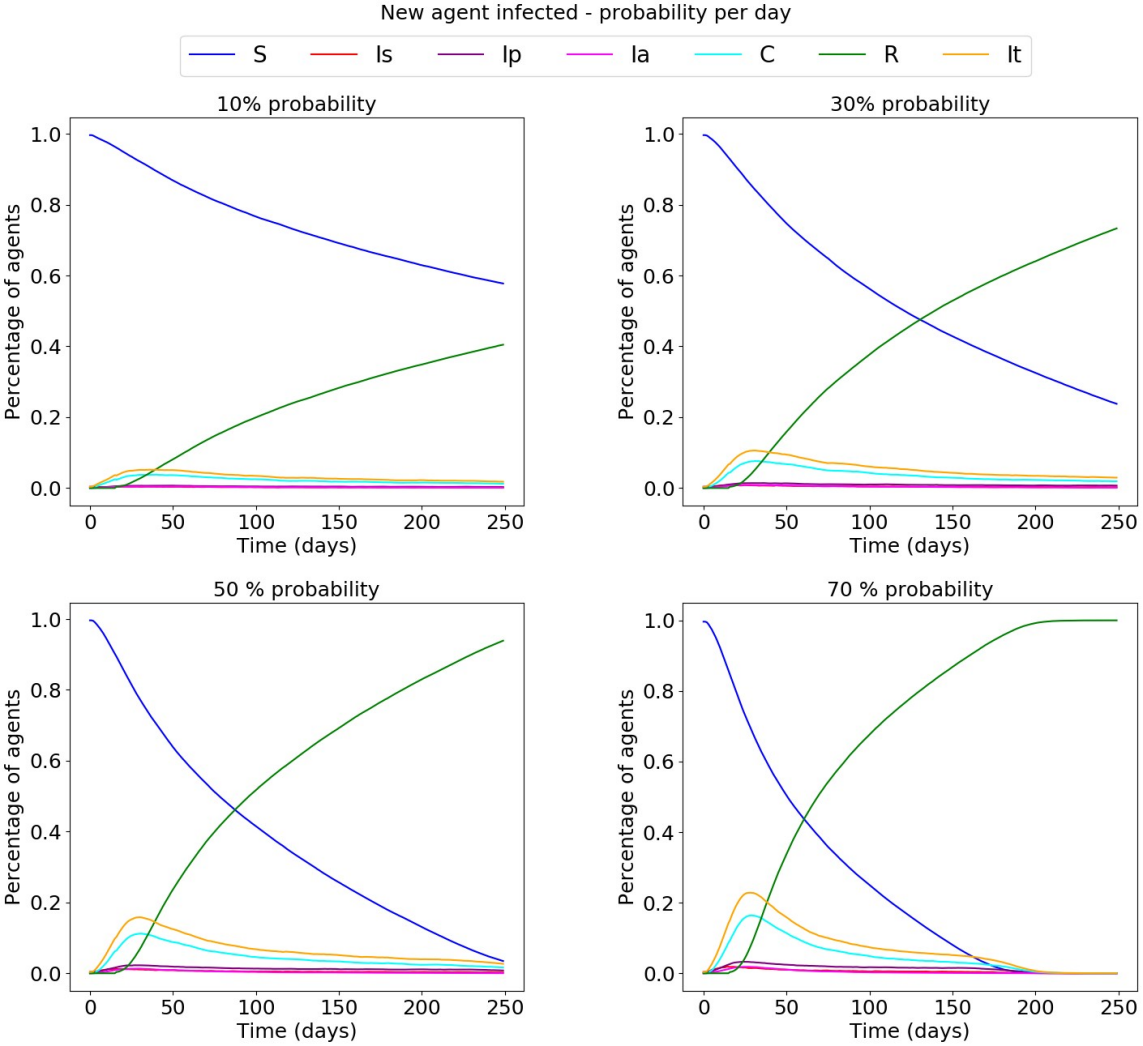
Percentage of nodes in an immunized stage (10, 30 and 60% on Sch1). The agents are selected based on different criteria: A) randomly, B) by its degree, C) by its clustering, and D) by its betweenness.

Fig 6 was obtained from the simulations of stage 5A (see Fig 2), where a percentage of immunized agents is assumed. The results were obtained using 10, 30, and 60% of immunized agents, either chosen randomly (Fig 6A), by its degree (Fig 6B), its clustering (Fig 6C), or its betweenness (Fig 6D). The results show that the best results are obtained when the immunized agents are chosen by either their degree or their betweenness. It is an expected result, since the degree measures the popularity of a node in the network. Thus, the more connected agents have a higher possibility of infecting others and being infected. On the other hand, betweenness measures the importance of a node for the network interconnection. Thus, the nodes with high betweenness are more susceptible to infection, since shorter paths pass through these nodes. Based on the percentages, if we immunized 60% of agents, the disease is contained. It is independent of how the agents are selected to be vaccinated.

## Discussion

Many studies have been conducted worldwide regarding COVID-19. Different countries have shown different levels of contagion at different times. Countries such as Australia, Japan, China and Germany, among others, have been able to continue or renew face-to-face classes in several academic institutions ranging from preschool to university levels. In Mexico, educational institutions were closed on March 20, 2020. Since then, they have remained closed except for a few cases. On January 11, 2021, schools in the states of Chiapas and Campeche (with records of very low contagion) were able to renew face-to-face activities. In other states, such as Veracruz, Tamaulipas, and Sinaloa, schools might open soon partially without the regular attendance of students. In this sense, the experience of going back to school in the context of the COVID-19 pandemic is new in Mexico. In this work, we assumed that massive COVID-19 tests would not be applied to students, parents, teachers, administrators, custodians, and others. Thus, it would be possible to have asymptomatic and presymptomatic students in the schools, which would not be detected until they infect others or show symptoms. Asymptomatic cases have been the subject of different studies [7, 34], and a consensus has not yet been reached on the percentage of asymptomatic patients who could exist within the sick population. In some cases, they are estimated to be 80% [35] of the patients and in others approximately 12 to 36% [46]. In this study, we aimed to simulate the best and worst scenarios in terms of control over the disease, based on the number of agents that do not present symptoms or present mild symptoms. It is not assumed that minors have the possibility of being less infectious, in accordance with studies that show that children have a contagion rate similar to adults [47]; however, there are reports of a lower contagion rate in the case of children [28]. It is important to note that in previous studies from schools, sampling was conducted once the protocols to mitigate the virus proliferation were established. Therefore, the contagious rate calculations could be affected by the effectiveness of the protocols and the reliability of the tests used [28, 29]. In contrast, in this work, only the student networks were considered. Nevertheless, having a sick close relative was comprised in the probability of contagion. Links with adults were not included, since it is assumed that returning to school coincides with a low level of contagion among adults, who have continued their activities with limited mobility.

Through this study, we attempt to contribute to the establishment of strategies that grant a low probability of producing a wave of contagion inside the schools, once returning to face-to-face activities. In stage 1, a spread of the virus among students is simulated without any mandatory sanitary measure; social distance and hygiene measures are randomly assumed to reproduce a state in which these are optionally adopted. At this stage, all of the schools show similar behavior (Fig 3B). At stage 2, the symptomatic agents are confined. If most agents do not present symptoms, it is difficult to control the disease spread by confining only the symptomatic agents. Stage 3 includes confining agents who show symptoms that begin mild (presymptomatic) and become severe, in addition to symptomatic agents. Fig 4A shows the evolution of the number of infected agents when the number of presymptomatic and asymptomatic agents change. Stage 4 includes enclosing the siblings of symptomatic agents. Fig 4B shows a comparison of stages 2 to 4. The results show that there is no appreciable difference in the total number of infected agents between the two stages. The measures simulated in these stages are similar to those initially established in several countries when COVID-19 cases were first found. In some countries, the contacts of infected patients were tracked, and massive COVID-19 testing was implemented, allowing the detection of presymptomatic and asymptomatic people. However, these initial measures were not sufficient. Based on Fig 4A and 4B, it is possible to say that other types of strategies are necessary to contain the spread of the disease.

It has been found, in different clinical studies, that the possibility of spreading the virus is not the same throughout the convalescence time [6, 48]. In stage 5, an approximation of the aforesaid trends in the viral load was incorporated such that on the days with a higher viral load, there is a greater possibility of infecting others and it declines as the days go on. Specifically, asymptomatic and presymptomatic cases could infect each day of their convalescence but not with the same probability, since it decreases while reaching the recovery state. Additionally, in this stage, symptomatic patients were confined the day they presented symptoms and not the day after, as in previous stages. It was assumed that the agents present symptoms the day after being infected; however, it could take between 1 and 3 days before they show them. Adding this dynamic did not make a significant difference compared to the previous stages.

To implement a strategy that addresses close interactions (which have been established as the principal form of contagion), we modified the network by eliminating links in two ways: i) dividing the classroom students into smaller groups (stage 6A) and ii) isolating the rooms, which implies avoiding all interactions with students from different classrooms, except for relatives (stage 6B). These stages engender a less dense network with a structure that is reduced to groups of nodes that are poorly interconnected. Fig 3C shows a comparison of stages 5 to 7, with stage 7 being the combination of stages 6A and 6B. From the results, it is evident that the separated strategies (6A and 6B) do not impact strongly the contagion dynamics. However, dividing the rooms and isolating them drives a significant decrease in the possible infections. This combination of strategies encompasses three situations in which government institutions around the world have requested to avoid back to school [30, 33, 49–51] 1) limited and closed spaces, 2) crowds, and 3) face-to-face interactions [28]. Specifically, dividing the classrooms into smaller groups and isolating the rooms allows avoiding crowds at the entrance of the classrooms and considerably decreases the face-to-face interactions [29].

Students are not separated from society; they are immersed in it. Thus, a student could get sick outside of the school aside from the family circle. To consider this fact, in stage 8, a daily probability that a random agent gets the virus is generated. Fig 5 shows how the number of infected agents increased with this daily probability. Stage 8 is a scenario of what could happen if the cases in adults increase while students are in schools. Currently, multiple vaccines for COVID-19 are available. However, vaccination of the world’s population is a complicated and distant task. The process is time-consuming, and new virus variations are appearing. In this respect, stage 5A preserves the conditions of previous stages and introduces a new state for the agents: immunized. Fig 6 shows how the virus spreads in the network when the immunized agents are generated using different criteria. It can be observed that immunity is more effective when the agents are chosen based on their degree or their betweenness.

### Recommendations based on simulations, literature, and field work

Based on the field work conducted to obtain the networks, we present some points that are worthwhile to consider and could not be included in the network. To avoid crowds, it is important to keep physical distance and reduce face-to-face interactions at common areas. Several of the institutions included in the study had two sessions, with 250 to 750 students per session. Having such a number of students in an entrance at the same time can have repercussions and increase the number of infections. Due to interactions outside of the schools, but necessary to enter the school, we simulate with a daily probability the increment of cases at schools (see Fig 5). In this sense, a staggered entrance of the students is recommended. The staggered arrival of students helps to avoid a large number of students meeting at the school access location. Additionally, it helps to avert a large number of interactions in public transportation. This avoidance would allow students from different classrooms to remain isolated from each other. Likewise, it is suggested that breaks be staggered and accompanied by sanitization of the area where students will have lunch, each time that a new group has its break. Similarly, a staggered exit is suggested to avoid crowds and interactions. In various studies, the authors mention the importance of contagion due to adults who are part of the staff of educational facilities [28, 29]. It is suggested to limit the interaction with parents and lower the number of staff members per day in addition to staggering their entry and exit.

### Limitations of the study

A first limitation comes from the fact that there are no data regarding infections in any of the participating schools to compare and properly adjust the variables. Thus, the simulations can give results that overestimate the number of cases that could occur. For this reason, many of the variables were established randomly. However, the parameters can be changed according to new findings. Another limitation comes from considering the student network to be a closed system, without accounting for adult family links or interactions with teachers. This study focuses on preventing the decision to go back to school from triggering the spread of the virus in minors who were in contingency for almost a year. Here, the practicality of executing the suggestions derived from the simulations is not accounted for, since simulations do not consider the particular context of the academic institutions from which the student networks were extracted. Therefore, the strategies obtained from this study must be discussed and contextualized in conjunction with the pertinent authorities at the social level, the school authorities and the parents before being implemented.

## Conclusion

Based on the simulations, it is possible to conclude that it is crucial to limit the interaction of students within an educational establishment once they are back to school. It is possible to see that getting groups smaller (stage 6A) or isolating different groups (stage 6B) is not the best strategy itself. However, the combination of these strategies shows the best results (Stage 7), because the number of infected agents decreased drastically (by 96.3% compared to Stage 5). It was also visible that even when the networks were composed of different types of students, the spread process was similar (Fig 3B). From previous work [52], it was possible to note that the ages and the locations of the students can influence the internal structures in the networks. It is significant to account for the social context of the different schools. For example, some schools have two class schedules, morning and afternoon. For these schools, the management of the entrance and exit of the students would be more intricate than for schools with a unique schedule. Because the simulations show that isolation and small groups are a good configuration, it can be extended for interactions outside the school spaces (for example, at the school entrance and on public transportation). In Fig 5, we simulate the infections due to the increments in cases outside the schools due to different factors using a daily probability. Then, it is adequate to implement strategies inside the schools and in the student’s transportation from home to school and vice versa. These simulations show similarities with strategies that have been implemented in countries that reopen their schools in the context of the current pandemic. Nevertheless, this work adds to the importance of adequate and limited interaction between students from different classrooms and the implications of changing the structure of the student network to maintain school activities with the least possible risk.

## Supporting information

S1 File Survey. The survey was used for obtaining the schools’ networks. This file is containing the English and Spanish versions of the questionary.
(PDF)

S2 File Adjacency matrices. Adjacency Matrices of directed networks used in this work.
(ZIP)

## References

[1] Amori M, Markhvida M, Hallegate S, Walsh B. Socio-Economic Impacts of COVID-19 on Household Consumption and Poverty. Economics of Disasters and Climate Change. 2020;4:453–459. doi: 10.1007/s41885-020-00070-3 PMC7376321 32838120

[2] Walker PGT, Whittaker C, Watson OJ, Baguelin M, Winskill P, Hamlet A, et al. The impact of COVID-19 and strategies for mitigation and suppression in low- and middle-income countries. Science. 2020. doi: 10.1126/science.abc0035 32532802 PMC7292504

[3] Milani F. COVID-19 outbreak, social response, and early economic effects: a global VAR analysis of cross-country interdependencies. Journal of Population Economics. 2021;34:223–252. doi: 10.1007/s00148-020-00792-4 PMC7437387 32839640

[4] Block P, Hoffman M, Raabe IJ, Dowd JB, Rahal C, Kashyap R, et al. Social network-based distancing strategies to flatten the COVID-19 curve in a post-lockdown world. Nature Human Behavior. 2020;4:588–596. doi: 10.1038/s41562-020-0898-6 32499576

[5] Prather KA, Wang CC, Schooley RT. Reducing transmission of SARS-CoV-2. Science. 2020. doi: 10.1126/science.abc6197 32461212

[6] He X, Lau EHY, Wu P, Deng X, Wang J, Hao X, et al. Temporal dynamics in viral shedding and transmissibility of COVID-19. Nature Medicine. 2020;26:672–675. doi: 10.1038/s41591-020-0869-5 32296168

[7] Yu C, Zhou M, Liu Y, Guo T, Ou C, Yang L, et al. Characteristics of asymptomatic COVID-19 infection and progression: A multicenter, retrospective study. Virulence. 2020;11:1006–1014. doi: 10.1080/21505594.2020.1802194 32722990 PMC7550018

[8] Ing AJ, Cocks C, Green JP. COVID-19: in the footsteps of Ernest Shackleton. Thorax. 2020;75:693–694. doi: 10.1136/thoraxjnl-2020-215091 32461231 PMC7402559

[9] Chen N, Zhou M, Dong X, Qu J, Gong F, Han Y, et al. Epidemiological and clinical characteristics of 99 cases of 2019 novel coronavirus pneumonia in Wuhan, China: a descriptive study. The Lancet. 2020. doi: 10.1016/S0140-6736(20)30211-7 32007143 PMC7135076

[10] Ferreti L, Wymant C, Kendall M, Zhao L, Nurtay A, Abeler-Dorner L, et al. CORONAVIRUS Quantifying SARS-CoV-2 transmission suggests epidemic control with digital contact tracing. Science. 2020. doi: 10.1126/science.abb6936 PMC7164555 32234805

[11] Medicine N. Using a real-world network to model localized COVID-19 control strategies. Firth, JA and Hellewell, J and Klepac, P and Kissler, S and CMMID COVID-19 Working group and Kucharski, AJ and Spurgin, LG. 2020. 10.1038/s41591-020-1036-8 32770169

[12] Jing QL, Liu MJ, Zhang ZB, Fang LQ, Yuan J, Zhang AR, et al. Household secondary attack rate of COVID-19 and associated determinants in Guangzhou, China: a retrospective cohort study. The Lancet. 2020. doi: 10.1016/S1473-3099(20)30471-0 32562601 PMC7529929

[13] Huang C, Wang Y, Li X, Ren L, Zhao J, Hu Y, et al. Clinical features of patients infected with 2019 novel coronavirus in Wuhan, China. The Lancet. 2020. doi: 10.1016/S0140-6736(20)30183-5 PMC7159299 31986264

[14] Pullano G, Valdano E, Scarpa N, Rubrichi S, Colizza V. Evaluating the effect of demographic factors, socioeconomic factors, and risk aversion on mobility during the COVID-19 epidemic in France under lockdown: a population-based study. The Lancet. 2020;2:e638–49. doi: 10.1016/S2589-7500(20)30243-0 33163951 PMC7598368

[15] van Doremalen N, Bushmaker T, Morrys DH, Phill M, Holbrook MG, Gamble A, et al. Aerosol and Surface Stability of SARS-CoV-2 as Compared with SARS-CoV-1. The New England Journal of Medicine. 2020. doi: 10.1056/NEJMc2004973 PMC7121658 32182409

[16] Zhang H, Chen R, Chen J, Chen B. COVID-19 Transmission Within a Family Cluster in Yancheng, China. Frontiers in Medicine. 2020;7:387. doi: 10.3389/fmed.2020.00387 32719808 PMC7349317

[17] Parri N, Lenge M, Buonsenso D. Children with COVID-19 in Pediatric Emergency Departments in Italy. The New England Journal of Medicine. 2020;383:187–190. doi: 10.1056/NEJMc2007617 32356945 PMC7206930

[18] Garazzino S, Montagnani C, Dona D, Meini A, Felici E, Vergine G, et al. Multicentre Italian study of SARS-CoV-2 infection in children and adolescents, preliminary data as at 10 April 2020. Euro Surveill. 2020;18:1–4. doi: 10.2807/1560-7917.ES.2020.25.18.2000600 32400362 PMC7219028

[19] Park SY, Kim YM, Yi S, Lee S, Na BJ, Kim CB, et al. Coronavirus Disease Outbreak in Call Center, South Korea. Emerging Infectious Diseases. 2020;26:1666–1670. doi: 10.3201/eid2608.201274 32324530 PMC7392450

[20] Hamner L, Dubbel P, Capron I, Ross A, Jordan A, Lee J, et al. High SARS-CoV-2 Attack Rate Following Exposure at a Choir Practice—Skagit County, Washington, March 2020. US Department of Health and Human Services. 2020;69:606–610. 10.15585/mmwr.mm6919e6 32407303

[21] Qian H, Miao T, Liu L, Zheng X, Luo D, Li Y. Indoor transmission of SARS-CoV-2. Indoor Air. 2020;0:1–7. doi: 10.1111/ina.12766 33131151

[22] Xie G. A novel Monte Carlo simulation procedure for modelling COVID‐19 spread over time. Science Reports. 2020;10:13120. doi: 10.1038/s41598-020-70091-1 32753639 PMC7403316

[23] Zakary O, Bidah S, Rachik M, Ferjouchia H. Mathematical Model to Estimate and Predict the COVID-19 Infections in Morocco: Optimal Control Strategy. Journal of Applied Mathematics. 2020;2020:1–13. doi: 10.1155/2020/9813926

[24] Zaplotnik Z, Gavrić A, Medic L. Simulation of the COVID-19 epidemic on the social network of Slovenia: Estimating the intrinsic forecast uncertainty. PlosOne. 2020;15:e0238090. doi: 10.1371/journal.pone.0238090 32853292 PMC7451520

[25] Chua MH, Cheng W, Goh SS, Kong J, Li B, Lim JYC, et al. Face Masks in the New COVID-19 Normal: Materials, Testing, and Perspectives. Research. 2020;2020:1–40. doi: 10.34133/2020/7286735 PMC7429109 32832908

[26] Chu DK, Akl EA, Duda S, Solo K, Yaacoub S, Schünemann HJ, et al. Physical distancing, face masks, and eye protection to prevent person-to-person transmission of SARS-CoV-2 and COVID-19: a systematic review and meta-analysis. The Lancet. 2020;395:1973–1987. doi: 10.1016/S0140-6736(20)31142-9 32497510 PMC7263814

[27] Leung NHL, Chu DKW, Shiu EYC, Chan KH, McDevitt JJ, Hau BJP, et al. Respiratory virus shedding in exhaled breath and efficacy of face masks. Nature Medicine. 2020;26:676–680. doi: 10.1038/s41591-020-0843-2 32371934 PMC8238571

[28] Macartney K, Quinn HE, Pillsbury AJ, Koirala A, Deng L, Winkler N, et al. Transmission of SARS-CoV-2 in Australian educational settings: a prospective cohort study. The Lancet. 2020. doi: 10.1016/S2352-4642(20)30251-0 32758454 PMC7398658

[29] Torres JP, Piñera C, De la Maza C, Logamarcino AJ, Simian D, Torres B, et al. Severe Acute Respiratory Syndrome Coronavirus 2 Antibody Prevalence in Blood in a Large School Community Subject to a Coronavirus Disease 2019 Outbreak: A Cross-sectional Study. Clinical Infectious Diseases. 2020. doi: 10.1093/cid/ciaa955 PMC7454451 32649743

[30] Stein-Zamir C, Abramson N, Shoob H, Libal E, Bitan M, Cardash T, et al. A large COVID-19 outbreak in a high school 10 days after schools’ reopening, Israel, May 2020. Euro Surveill. 2020;25:1–5. doi: 10.2807/1560-7917.ES.2020.25.29.2001352 PMC7384285 32720636

[31] of Education New Zealand M. Health and safety in licensed early learning services and kohanga reo for COVID-19; 2020.

[32] Moghadas SM, Fitzpatrick MC, Sah P, Pandey A, Shoukat A, Singer BH, et al. The implications of silent transmission for the control of COVID-19 outbreaks. PNAS. 2020;117:17513–17515. doi: 10.1073/pnas.2008373117 32632012 PMC7395516

[33] Organization WH. Checklist to support schools re-opening and preparation for COVID-19 resurgences or similar public health crises; 2020.

[34] Zheng X, Luo S, Sun Y, Han M, Liu J, Sun L, et al. Asymptomatic patients and asymptomatic phases of Coronavirus Disease 2019 (COVID-19): a population-based surveillance study. National Science Review. 2020;7:1527–1539. doi: 10.1093/nsr/nwaa141 PMC7337770 34676080

[35] Group CSA. COVID-19 Scientific Advisory Group Rapid Response Report.

[36] Yazdanpanah F, Hamblin MR, Rezaei N. The immune system and COVID-19: Friend or foe? Life Sciences. 2020;256:1–5. doi: 10.1016/j.lfs.2020.117900 32502542 PMC7266583

[37] Chowdhury MA, Hossain N, Kashem MA, Shahid A, Alam A. Immune response in COVID-19: A review. Journal of Infection and Public Health. 2020;13:1619–1629. doi: 10.1016/j.jiph.2020.07.001 32718895 PMC7359800

[38] Stadnytskyi V, Bax CE, Bax A, Anfinrud P. The airborne lifetime of small speech droplets and their potential importance in SARS-CoV-2 transmission. PNAS Latest Articles. 2020. doi: 10.1073/pnas.2006874117 32404416 PMC7275719

[39] Li Y, Ren B, Peng X, Hu T, Li J, Gong T, et al. Saliva is a non-negligible factor in the spread of COVID-19. Molecular Oral Microbiology. 2020;35:141–145. doi: 10.1111/omi.12289 32367576 PMC7267240

[40] Xu R, Cui B, Duan X, Zhang P, Zhou X, Yuan Q. Saliva: potential diagnostic value and transmission of 2019-nCoV. International Journal of Oral Science. 2020;12:1–6. doi: 10.1038/s41368-020-0080-z 32300101 PMC7162686

[41] Ghinai I, Woods S, Ritger KA, McPherson TD, Black SR, Sparrow L, et al. Community Transmission of SARS-CoV-2 at Two Family Gatherings—Chicago, Illinois, February-March 2020. US Department of Health and Human Services/Centers for Disease Control and Prevention. 2020;69:446–450. 10.15585/mmwr.mm6915e1 PMC7755060 32298246

[42] Panorama Sociodemografico del Municipio de Merida, el Estado de Yucatan y Nacional.

[43] Huerta-Quintanilla R, Canto-Lugo E, Viga-de Alva D. Modeling Social Network Topologies in Elementary Schools. PlosOne. 2013;8:e55371. doi: 10.1371/journal.pone.0055371 PMC3567059 23408976

[44] Lounis M, Bagal DK. Estimation of SIR model’s parameters of COVID‐19 in Algeria. Bulletin of the National Research Centre. 2020;44:1–6. doi: 10.1186/s42269-020-00434-5 33100825 PMC7570398

[45] Flaxman S, Mishra S, Gandy A, Unwin HJT, Coupland H, Mellan TA, et al. Report 13: Estimating the number of infections and the impact of non-pharmaceutical interventions on COVID-19 in 11 European countries; 2020.

[46] Yanes-Lane M, Winters N, Fregonese F, Bastos M, Periman-Arrow S, Campbell JR, et al. Proportion of asymptomatic infection among COVID-19 positive persons and their transmission potential: A systematic review and meta-analysis. PlosOne. 2020;15:1–21. doi: 10.1371/journal.pone.0241536 33141862 PMC7608887

[47] Bi Q, Wu Y, Mei S, Ye C, Zou X, Zhang Z, et al. Epidemiology and transmission of COVID-19 in 391 cases and 1286 of their close contacts in Shenzhen, China: a retrospective cohort study. The Lancet. 2020;20:911–919. doi: 10.1016/S1473-3099(20)30287-5 32353347 PMC7185944

[48] Cevik M, Tate M, Lloyd O, Maraolo AE, Shafers J, Ho A. SARS-CoV-2, SARS-CoV, and MERS-CoV viral load dynamics, duration of viral shedding, and infectiousness: a systematic review and meta-analysis. The Lancet. 2021;2:e13–e22. doi: 10.1016/S2666-5247(20)30172-5 33521734 PMC7837230

[49] Ministry of Education SS Cultural, Japan T. New School Lifestyle—COVID-19 Infection Control Manuals and Guidelines for Schools; 2020.

[50] Australian Government Departament of Education S, Employment. China’s education arrangements during COVID-19 pandemic period; 2020.

[51] Chen P, Mao L, Nassis JP, Harmer P, Ainsworth BE, Li F. Returning Chinese school-aged children and adolescents to physical activity in the wake of COVID-19: Actions and precautions. Journal of Sport and Health Science. 2020;9:322–324. doi: 10.1016/j.jshs.2020.04.003 32325023 PMC7154517

[52] Hernaández-Hernández AM, Viga-de Alva D, Huerta-Quintanilla R, Canto-Lugo E, Laviada-Molina H, Molina-Segui F. Friendship Concept and Community Network Structure among Elementary School and University Students. PlosOne. 2016;11:e0164886. doi: 10.1371/journal.pone.0164886 PMC5070781 27760171

