# Supplementary material for: Managing school interaction networks during the COVID-19 pandemic: Agent-based modeling for evaluating possible scenarios when students go back to classrooms
Source: PLoS One. 2021 Aug 18;16(8):e0256363. doi: 10.1371/journal.pone.0256363 (PMC8372954; doi:10.1371/journal.pone.0256363)
Supplement: S1 File — The survey was used for obtaining the schools’ networks. This file is containing the English and Spanish versions of the questionary. (PDF) [file pone.0256363.s001.pdf]

SCHOOL NETWORKS SURVEY

English Version

I. Personal Data

1. Please indicate your age on years ___________________
2. Please indicate your sex   __men   _women
3. How many brothers and systers do you have? _______________

II. Social data

1. Make a list of words (6 words tops) to indicate what is a friend for you
2. Make a list of words (6 words tops) to indicate why someone is NOT your friend
3. Whom of the students of the school are your friends?
4. With which students do you not get along or have a bad relationship?
5. Whom of the students of the school are your biological brothers or sisters?
6. Whom of the students of the school are your biological cousins?
7. Whom of the students of the school do you usually get along for studying for exams or for doing homework?

Spanish Version

I. Datos personales

1. Por favor indica tu edad en años _____________
2. Por favor indica tu sexo ___Hombre   ____Mujer
3. ¿Cuántos hermanos y hermanas tienes? _________

II. Datos sociales

1. ¿Cuáles estudiantes de la escuela son tus amigos?
2. ¿Con cuales estudiantes tienes una relación poco cordial o una mala relación?
3. ¿Quiénes de los estudiantes de la escuela son tus hermanos/hermanas biológicas?
4. ¿Quiénes de los estudiantes de la escuela son tus primos/primas biológicas?
5. ¿Con cuáles estudiantes te reúnes para estudiar para exámenes o hacer tareas?
